# Physiological Effects of Oxygen

**Published:** 1873-05

**Authors:** 


### ARTICLE VIII.

### *Physiological Effects of Oxygen.*

M. Bert, a French chemist, has been conducting a series of experiments with a view to test the properties of oxygen gas as a stimulant. In a paper read before the Academy, at Paris, appears the following practical suggestions :

" Animals," says the author, " were confined in superoxygenated air, the pressure of which was over 2½ atmospheres. Here un unforseen result was met with. When the pressure was raised to four or five atmospheres, the animal gave signs of suffering and fell into convulsions, which were repeated up to the time of its death (in less than half an hour.) These violent effects could not be due simply to pressure, for in common air the animals bore a pressure of eight or nine atmospheres without inconvenience. They are due to a large proportion of oxygen, which, entering the blood, acted poisonously. To produce the same results by simply using air, it would be necessary to exceed 14 atmospheres. Oxygen, therefore, when its proportion in the blood is notably increased, acts as a poison.

At a later period M. Bert was able to apply a pressure of 20 atmospheres. With common air, the convulsions due to poisoning, by oxygen appeared at 15 atmospheres.

Still M. Bert thinks (and he gives proof of it) the injurious action of oxygen must not be supposed to commence there. It begins to act thus at six atmospberes, or, as more refined experiments may yet show, at even lower pressures.

The benefit derived from use of compressed air-baths on the one side, and many of the accidents that happen to workers in mines, diving-bells, etc., on the other, appear to be due, in great part, to the introduction into the system of a quantity of oxygen greater than in the normal state; and it is with oxygen as with other poisons, small doses of which may be salutary.

If æronauts, who are stopped in their upward course, not by failure in the baloon, but by the impossilility of living, wished to soar higher, they might accomplish this if they took with them a supply of oxygen to which they could have recourse when the air became too rare. The mechanical arrangements for breathing this conveniently can be easily conceived. On the the other hand, the industries which subject workmen to pressures over five or six atmospheres, entail suffering and death; which evils might be remedied if there were substituted for the air a mixture of air and nitrogen, calculated so that the pressure of the oxygen should not exceed a certain low limit.—*Druggists' Circular.*

